# Genomic Analysis of Avian Infectious Bronchitis Viruses Recently Isolated in South Korea Reveals Multiple Introductions of GI-19 Lineage (QX Genotype)

**DOI:** 10.3390/v13061045

**Published:** 2021-05-31

**Authors:** Hyuk-Chae Lee, Sol Jeong, Andrew Y. Cho, Kyu-Jik Kim, Jun-Young Kim, Dam-Hee Park, Hyun-Jin Kim, Jung-Hoon Kwon, Chang-Seon Song

**Affiliations:** 1 College of Veterinary Medicine, Konkuk University, 120, Neungdong-ro, Gwangjin-gu, Seoul 05029, Korea; big4103@gmail.com (H.-C.L.); soljeong492@gmail.com (S.J.); 7rewcho@gmail.com (A.Y.C.); zidanov31@gmail.com (K.-J.K.); okddr11@gmail.com (J.-Y.K.); pdh03079@gmail.com (D.-H.P.); scv1188@gmail.com (H.-J.K.); 2 College of Veterinary Medicine, Kyungpook National University, 80, Daehak-ro, Buk-gu, Daegu 41566, Korea

**Keywords:** IBV, avian coronavirus, surveillance, South Korea, multiple introductions, phylogenetic inference

## Abstract

Infectious bronchitis virus (IBV) was first identified in the 1930s and it imposes a major economic burden on the poultry industry. In particular, GI-19 lineage has spread globally and has evolved constantly since it was first detected in China. In this study, we analyzed *S1* gene sequences from 60 IBVs isolated in South Korea. Two IBV lineages, GI-15 and GI-19, were identified in South Korea. Phylogenetic analysis suggested that there were six distinct subgroups (KM91-like, K40/09-like, and QX-like I to IV) of the South Korean GI-19 IBVs. Among them, QX-type III and IV subgroups, which are phylogenetically different from those reported in South Korea in the past, accounted for more than half of the total. Moreover, the phylogeographic analysis of the QX-like subgroups indicated at least four distinct introductions of GI-19 IBVs into South Korea during 2001–2020. The efficacy of commercialized vaccines against the recently introduced QX-like subgroups should be verified, and continuous international surveillance efforts and quarantine procedures should be enhanced to prevent the incursion of viruses.

## 1. Introduction

Infectious bronchitis virus (IBV), first isolated in the 1930s, is the etiological agent of an acute and highly contagious respiratory, renal, and genital disease that affects chickens of all ages and imposes a major economic burden on the poultry industry [1]. IBV, which belongs to the family *Coronaviridae*, genus *Gammacoronavirus*, is a single-stranded, positive-sense RNA virus [2]. IBV is an enveloped virus with a genome of approximately 27 kb that displays the following gene organization: 5′UTR-1a/1ab-S-3a-3b-E-M-5a-5b-N-3′UTR [3]. The spike (S) glycoprotein is proteolytically cleaved into two subunits, S1 and S2. S1 plays a major role in cell attachment, tissue tropism, and serotype determination, while the S2 subunit anchors the spike protein into the viral membrane [4,5]. The high mutation rate of the *S1* gene during IBV replication poses a continuous challenge in the control of infectious bronchitis viruses [6,7]. Due to the roles of the *S1* gene in immunity and virus diversity, the genetic classification and evolutionary analysis of IBV have generally focused on the this gene [2,8].

Recently, Valastro et al. [8] define the *S1* phylogeny-based classification system for the harmonized classification of IBV strains. In this classification system, global IBV strains are divided into 6 genotypes (GI–GVI) and 32 distinct lineages (GI-1 to GI-27, GII-1, GIII-1, GIV-1, GV-1 and GVI-1). Most IBV lineages are confined to specific geographic regions, and some countries possess unique lineages. In contrast, the GI-1, GI-13, GI-16 and GI-19 lineages are widely distributed [8]. The GI-1 and GI-13 (also called Mass and 793B types) are commonly found in various countries due to the use of vaccines derived from the lineages. The GI-19 lineage, also called QX-like, has been identified China, Japan, Korea, Europe, Russia, Africa, and the Middle East, causing severe nephritis and ‘false layer’ syndrome [8].

IBV was first reported in South Korea in 1986, following the observation of certain respiratory symptoms in chickens. It was named Korean group I (K-I) and was later classified as the GI-15 lineage [8,9]. In 1990, a nephropathogenic strain of IBV, now known as the Korean group II (K-II) subgroup KM91-like strain [10], caused severe damage to the chicken industry in South Korea. The QX genotype, which was initially isolated in China in 1993, is now widespread globally and was introduced to South Korea around 2002–2003. This strain is now known as the K-II subgroup QX-like strain [6,11]. Novel recombinants of the KM91-like and QX-like strains, here after ‘K40/09-like’, emerged in 2009 and these are the currently prevailing strains in South Korea [12]. According to the IBV *S1* gene classification system suggested by Valastro et al. [8], all K-II subgroups (KM91-like, K40/09-like, and QX-like) and QX genotypes belong to the GI-19 lineage.

As the introduction and emergence of new IBV variants may impede the disease control efficacy of the vaccines against this virus, it is important to monitor the prevalence and genetic characteristics of IBV [6,13]. Here, we studied recently isolated IBVs in South Korea and sequenced the *S1* subunit of the spike gene to classify IBV lineages and perform phylogenetic analysis.

## 2. Materials and Methods

### 2.1. Sample Collection and Virus Isolation

Viruses were isolated from chicken carcasses that suspected IBV infection in South Korea between 2016 and 2020 (Supplementary Table S1). The trachea, kidney, and cecal tonsils of the chickens were homogenized and diluted to 10% (*w*/*v*) with phosphate-buffered saline containing 400 mg/mL gentamicin. The supernatants of homogenized tissue samples were filtered using a 0.45 Minisart syringe filter (Sartorius, Göttingen, Germany) and propagated in 10-day-old specific pathogen-free embryonated chicken eggs at 37 °C for 72 h [12]. The allantoic fluids were then harvested and viral RNA was extracted from the harvested allantoic fluid using an RNeasy Mini Kit (Qiagen, Hilden, Germany) according to the manufacturer’s instructions. To detect IBV, the 5′-UTR region of infectious bronchitis virus was amplified using real-time reverse transcription-PCR (rRT-PCR) as previously described [14].

### 2.2. Sequencing and Phylogenetic Analysis of Isolates

The *S1* gene of the IBV isolates was amplified using a OneStep RT-PCR Kit (Qiagen) according to the manufacturer’s instructions, with previously described primer sets [15]. The PCR products were purified using a GeneJET Gel Extraction Kit (Thermo Fisher Scientific, Waltham, MA, USA) and sequenced by Sanger sequencing services (Macrogen Co., Ltd., Seoul, South Korea). The sequences obtained in this study were aligned with the prototype strain of each lineage of IBV and the reference strains [8] using MAFFT v7.308 (https://mafft.cbrc.jp/alignment/software/, accessed on 11 May 2021). Reference strains with over 90% sequence identity with the isolates were selected (Table S2) using the BLASTn tool (https://blast.ncbi.nlm.nih.gov/, accessed on 11 May 2021). The maximum-likelihood phylogenetic tree for alignment was constructed using RAxML, a general time-reversible (GTR) nucleotide model and gamma distribution with 1000 rapid bootstrap replicates [16]. The tree was finally visualized and annotated using Interactive Tree of Life v1.0 (https://itol.embl.de/, accessed on 11 May 2021).

### 2.3. Phylogeographic and Time-Scaled Phylogenetic Analysis of Isolates Clustered into the South Korean QX-Like IBV Subgroups

The presence of recombinant strains was determined using RDP4 [17] and recombinants detected with more than two methods with a significance value lower than 10^−5^ (*p*-value *<* 10^−5^) were excluded [18]. The final data set consisted of 181 taxa, which were coded into three geographic locations: South Korea (*n* = 56), China (*n* = 78), and Europe (*n* = 47). Bayesian time-scaled phylogenetic estimation was performed using BEAST v.1.8.4 software [19]. Nucleotide substitutions and clock models were selected using the Bayesian information criterion calculated using jModelTest [20]. The Gaussian Markov random field Bayesian skyride coalescent tree was constructed using a GTR substitution model with gamma site heterogeneity and an uncorrelated relaxed clock. We reconstructed the ancestral location state and estimated the asymmetric viral exchanges between locations by non-reversible continuous-time Markov chain (CTMC) model in discrete space using the BEAST package [21]. We also estimated the rate and number of migrations between locations (Markov jump) using stochastic mapping techniques implemented in BEAST [22]. Monte Carlo Markov chains were run for four chains, each with 50 million steps and the parameters and trees sampled every 5000 steps. The resulting log and tree files were combined with LogCombiner v1.10.4 (https://beast.community/logcombiner, accessed on 11 May 2021) after 10 per cent burn-in yielding a total 36,004 parameter states and posterior trees. The results were analyzed using Tracer 1.7.1, and accepted only if the effective sample size was greater than 200, and the convergence and mixing were adequate [18]. The maximum clade credibility (MCC) tree was summarized using a TreeAnnotator (BEAST package) and visualized using FigTree v.1.4.4 (http://tree.bio.ed.ac.uk/software/figtree/, accessed on 11 May 2021).

## 3. Results

**Figure 1:**
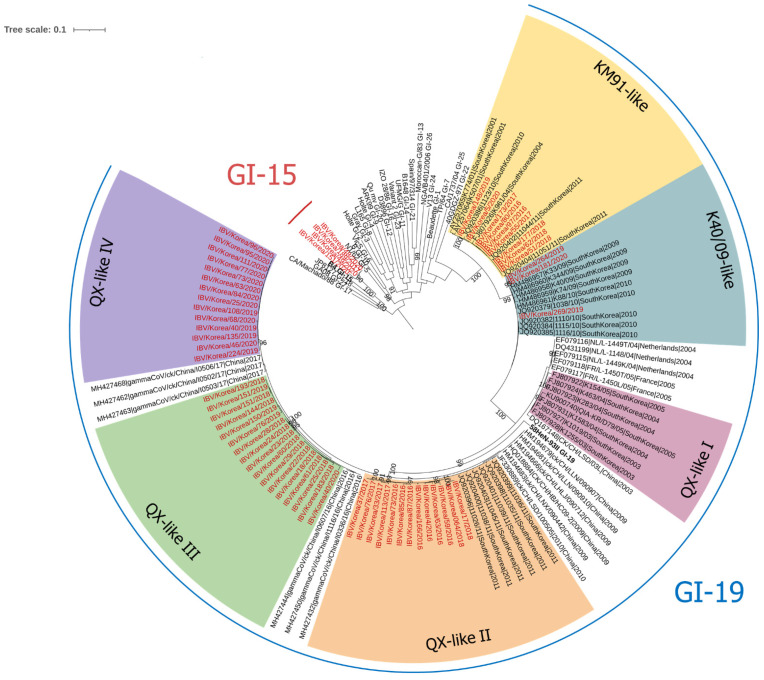
Maximum-likelihood phylogenetic tree of the IBV spike 1 subunit (*S1*) gene. Each prototype of the lineages (GI-15; B4 and GI-19; 58HeN-93II) are in bold font. Taxa colored red are the IBVs isolated in this study. The six subgroups of the South Korean GI-19 IBV strain are annotated with different background colors.

Sixty IBVs were isolated from 2016 to 2020 (Figure 1 and Table S1). Four isolates (IBV/South Korea/189/2017, IBV/South Korea/48/2020, IBV/South Korea/149/2020, and IBV/South Korea/151/2020) were closely clustered into the GI-15 lineage, and the remaining isolates were closely clustered into the GI-19 lineage. Six distinct subgroups of the South Korean GI-19 IBVs (supported by >99 bootstrap values) were also identified in the tree (KM91-like, K40/09-like, and QX-like I to IV).

The KM91-like and K40/09-like subgroups were found to be isolated only from South Korea. However, the QX-like I subgroup clustered with IBVs isolated in China, South Korea, France, and the Netherlands in 2003, 2004, and 2005. The QX-like II subgroup consisted of IBVs isolated in China and South Korea in 2009, 2010, and 2011 [13]. However, the QX-like III and IV subgroups clustered with IBVs reported in China in 2016 and 2017 [23]. Of the 56 analyzed isolates, 9 belonged to the KM91-like subgroup, 3 belonged to the K40/09-like subgroup, 13 belonged to the QX-like II subgroup, 17 belonged to the QX-like III subgroup, and 14 belonged to the QX-like IV subgroup. However, no viruses isolated in this study were clustered into the QX-like I subgroup.

The nucleotide and amino acid sequence identities of the *S1* gene between the South Korean GI-19 IBVs (KM91-like, K40/09-like, and QX I to IV) were calculated (Table 1). The nucleotide and amino acid identities between the KM91-like and K40/09-like subgroups were 91.8–93.1% and 88.8–91.1%, respectively. The four QX-like subgroups showed relatively low nucleotide and amino acid identities (<90%) with the prevailing South Korean IBV subgroups, KM91-like and K40/09-like. Meanwhile, the nucleotide and amino acid identities between the newly introduced QX-like subgroups (QX-like II, III, and IV) were 95.8–99.9% and 93.6–99.4%, respectively, whereas QX-like I showed relatively low identities with these subgroups (<96%).

We estimated viral movement between regions of QX-like subgroups (I-IV) using time-scaled phylogeography. The *S1* gene sequences were aligned with the reference strains (Supplementary Table S2) in GenBank (https://www.ncbi.nlm.nih.gov/genbank/ accessed on 11 May 2021).

In the MCC tree, all four distinct South Korean QX-like IBV subgroups shared a common ancestral node with a high posterior probability support (>70%, Table 2). IBVs isolated in South Korea during 2003–2004 were clustered into the QX-like I subgroup. The common ancestral location of the QX-like I subgroup was estimated to be China, but the posterior probability was relatively low (77.55%). However, the QX-like I subgroup has not been detected in South Korea since 2006, and IBVs isolated in South Korea during 2016–2020 consisted of phylogenetically distinct clusters (QX-like II to IV). The QX-like II subgroup was first reported in South Korea in 2010 [24] and was identified in 2016, 2017, and 2018 in this study. QX-like III and IV subgroups were first reported in this study. The QX-like III subgroup has been observed in South Korea since 2018, but the QX-like IV subgroup was first identified in South Korea in 2019. The ancestral location of QX-like II to IV subgroups was estimated to be China, with a high posterior probability (> 95%, Table 2), and the QX-like III and IV subgroups were clustered together into the “China A sublineage” [23]. The mean number of viral migrations from China to South Korea in 36,004 posterior trees was computed and estimated to be 4.48 (95% credible interval: 4–6).

**Figure 2:**
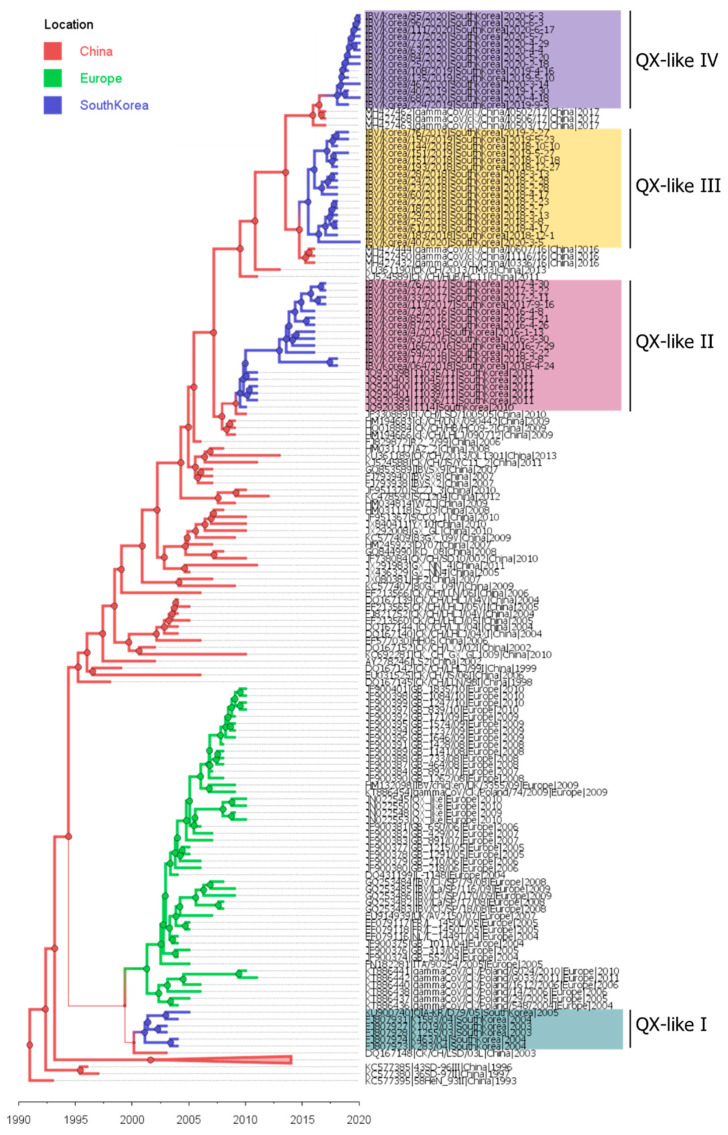
The maximum clade credibility tree of the isolates clustered into the South Korean QX-like IBV subgroups. The color of the branches and nodes (circle) indicates the isolate’s location of origin (red, China; green, Europe; blue, South Korea). The branch weight and size of the node depend on the location probability. Taxa divided by background color indicate the QX-like subgroups, QX-like IV to I, in order from the top.

We also estimated the time of virus introduction by calculating the time to the most recent common ancestor (tMRCA) of each South Korean QX-like IBV subgroup (Table 2). The mean tMRCA of the QX-like I to IV subgroups was estimated as February 2001 (QX-like I), July 2009 (QX-like II), July 2015 (QX-like III), and February 2018 (QX-like IV). These results indicated that at least four independent virus introductions into South Korea occurred from 2001 to 2020 (Figure 2, Table 2).

## 4. Discussion

A previous study conducted in Europe showed a rapid spread of newly introduced viruses and displacement of the dominant lineage. A single introduction event in early 2000 was followed by the establishment and rapid local expansion of GI-19 IBVs [18]. In previous phylogenetic studies of GI-19 IBVs in South Korea, three distinct subgroups, KM91-like, K40/09-like, and QX-like, were identified. Two of these subgroups (KM91-like and K40/09-like) have only been reported in South Korea, while the QX-like subgroup was speculated to have originated abroad [6,11,12]. However, at least four distinct QX-like subgroups were identified in South Korea in this study (Figure 1). In addition, through phylogeographical analysis, we found that viruses of the QX-like subgroups were introduced into South Korea at different times (Figure 2). Reference strains close to the QX-like III and QX-like IV subgroups belong to the same cluster, the “China A sublineage”, in China [23]. It seems that viruses of the same sublineage were introduced into South Korea twice at different time points. As the sequences of IBV isolated in other countries are not fully available or are relatively short, the routes of introduction of GI-19 IBVs into South Korea are unclear. The import of the unheated poultry product from highly pathogenic avian influenza (HPAI) outbreak countries, including China, have been prohibited in South Korea. However, as is the case for the HPAI virus detection in Europe, Japan, and North America [25,26,27,28], poultry products and raw poultry meat illegally imported by airline passengers are suspected to be the source of IBV introduction into South Korea.

Vaccination to prevent IBV infections is widely used globally [2,6]. However, there are some limitations, such as poor cross-protection between different serotypes and vaccine failure caused by antigenic drift of the viruses [7,12,13]. In South Korea, live attenuated vaccines using the KM91-like origin [29] and the K40/09-like origin [30] strains have been developed and successfully commercialized. Moreover, a vaccine originating from the K40/09 strain showed effective cross-protection against both KM91 and QX-like I viruses [30]. However, the nucleotide and amino acid sequence identities of the QX-like I subgroup were quite different from those of the other QX-like subgroups (Table 1). Therefore, efficacy studies of commercialized vaccines for viruses of the QX-like II to IV subgroups and vaccine development to prevent the spread of the newly identified QX-like subgroup are needed.

Here, we identified that two lineages of IBV (GI-15 and GI-19) and the five distinct subgroups of GI-19 (KM91-like, K40/09-like, QX-like II, III, and IV) are co-circulating and evolving in South Korea. Although the QX-like I subgroup was not detected in this study, more large-scale studies are needed to confirm the disappearance of this subgroup. We also suggest the possibility of the occurrence of several incursions of genetically distinct GI-19 IBVs in South Korea, which may have led to the diversification of the genetic pool of IBVs in South Korea.

The efficacy of commercialized vaccines against new GI-19 strains should be verified, and continuous international surveillance efforts and enhanced quarantine procedures should be performed to prevent the incursion of novel viruses. The IBV surveillance needs to be more focused on genomic analysis, not just identification, for identifying the introduction of novel lineages and the changes of dominant lineages.

## Figures and Tables

**Table 1:** Nucleotide^1^ and amino acid identities of the *S1* gene ^1^ between the South Korean GI-19 IBVs.

Subgroups	KM91-Like	K40/09-Like	QX-Like I	QX-Like II	QX-Like III	QX-Like IV
	Amino acid identity (%)					
KM91-like	**95.6–100 ^2^**	**88.8–91.1**	**83.5–85.7**	**82.1–86.1**	**83.2–85.9**	**83.4–86.1**
	97.2–100 ^3^					
K40/09-like	91.8–93.1	**96.6–99.6**	**88.4–90.9**	**86.3–90.1**	**86.8–90.3**	**87–89.9**
		98.3–99.9				
QX-like I	83.9–85.6	89.4–90.8	**95.6–99.6**	**91.4–94.9**	**92.8–94.8**	**92.6–94.8**
			97.7–99.8			
QX-like II	83.9–85.8	87.6–89.4	93.7–95.8	**95.2–99.6**	**93.6–97.9**	**93.8–98.1**
				97.5–99.8		
QX-like III	83.6–85.4	87.5–88.9	94.1–95.6	95.8–97.9	**96.4–99.8**	**96.6–99.4**
					98.0–99.9	
QX-like IV	84.0–85.5	87.4–88.6	94–95.2	95.9–97.8	97.5–98.9	**98.1–100**
						98.9–99.9
	Nucleotide identity (%)					

Top right, amino acid identity (%); bottom left, nucleotide identity (%). Bold text indicates amino acid identity. Nonbold text indicates nucleotide identity. ^1^ Partial nucleotides of the *S1* gene (1575–1598 bp) were compared. ^2^ Amino acid identity (%). ^3^ Nucleotide identity (%).

**Table 2:** tMRCA and posterior probability of the clades observed in the MCC tree of Figure 2.

Subgroups	tMRCA ^1^ (95% BCI ^2^)	Posterior Probability ^3^	Ancestral Location ^4^ (Probability)
QX-like I	13 February 2001 (23 October 1999~27 April 2002)	0.72	China (77.55%)
QX-like II	9 July 2009 (15 January 2009~6 December 2009)	0.94	China (95.49%)
QX-like III	6 July 2015 (4 March 2014~4 May 2016)	0.98	China (99.99%)
QX-like IV	10 February 2018 (22 August 2017~20 July 2018)	1	China (99.45%)

^1^ tMRCA: time to the most recent common ancestor. ^2^ 95% BCI: 95% Bayesian credible intervals. ^3^ The posterior probability supporting node for each subgroup was estimated by Bayesian phylogenetic analysis. ^4^ The location of the most recent ancestral node before introducing into South Korea and their probability were estimated by Bayesian phylogeography.

## Data Availability

The data presented in this study are available on request from the corresponding author.

## Supplementary Materials

The following are available online at https://www.mdpi.com/article/10.3390/v13061045/s1, Table S1: Backgrounds of IBV isolates analyzed in this study. Table S2: GI-19 reference strains used in this study.
