# Supplementary material for: Genomic Analysis of Avian Infectious Bronchitis Viruses Recently Isolated in South Korea Reveals Multiple Introductions of GI-19 Lineage (QX Genotype)
Source: Viruses. 2021 May 31;13(6):1045. doi: 10.3390/v13061045 (PMC8228071; doi:10.3390/v13061045)
Supplement: Supplementary file 1 [file viruses-13-01045-s001.zip › viruses-1236685-supplementary.pdf]

viruses

Supplementary

Table S1. Backgrounds of IBV isolates in this study.

Virus	Nation	Date	Host	Age	Clinical signs	Genotype	Accession number
IBV/Korea/151/2020	South Korea	2020-09-15	Broiler	-	respiratory signs	GI-15	MW877606
IBV/Korea/49/2020	South Korea	2020-03-17	Broiler	29days	respiratory signs	GI-15	MW877607
IBV/Korea/189/2017	South Korea	2017-11-15	Broiler	26 days	respiratory signs	GI-15	MW877608
IBV/Korea/48/2020	South Korea	2020-03-17	Broiler	29 days	respiratory signs, colibacillosis	GI-15	MW877609
IBV/Korea/40/2020	South Korea	2020-03-05	Broiler	-	air sacculitis	GI-19	MW877610
IBV/Korea/68/2020	South Korea	2020-04-18	Broiler	21 days	nephritis	GI-19	MW877611
IBV/Korea/224/2019	South Korea	2019-09-03	Broiler	30 days	nephritis, urate deposition	GI-19	MW877612
IBV/Korea/63/2020	South Korea	2020-04-04	Broiler	29 days	nephritis, urate deposition	GI-19	MW877613
IBV/Korea/77/2020	South Korea	2020-05-07	Broiler	25 days	colibacillosis, nephritis	GI-19	MW877614
IBV/Korea/95/2020	South Korea	2020-06-03	Broiler	21 days	respiratory signs, nephritis	GI-19	MW877615
IBV/Korea/111/2020	South Korea	2020-06-17	Broiler	30 days	urate deposition	GI-19	MW877616
IBV/Korea/96/2020	South Korea	2020-06-03	Broiler	21 days	colibacillosis	GI-19	MW877618
IBV/Korea/135/2019	South Korea	2019-05-10	Broiler	29 days	colibacillosis, urate deposition	GI-19	MW877619
IBV/Korea/84/2020	South Korea	2020-05-20	Broiler	-	no exact record	GI-19	MW877620
IBV/Korea/25/2020	South Korea	2020-02-18	Broiler	18 days	air sacculitis	GI-19	MW877621
IBV/Korea/46/2020	South Korea	2020-03-14	Broiler	-	nephritis, urate deposition	GI-19	MW877622
IBV/Korea/73/2020	South Korea	2020-04-29	Broiler	-	colibacillosis	GI-19	MW877623
IBV/Korea/108/2019	South Korea	2019-04-16	Broiler	32 days	air sacculitis	GI-19	MW877624
IBV/Korea/40/2019	South Korea	2019-01-30	Broiler	29 days	colibacillosis	GI-19	MW877625
IBV/Korea/183/2018	South Korea	2018-12-01	Broiler	16 days	nephritis	GI-19	MW877626
IBV/Korea/29/2018	South Korea	2018-03-13	Broiler	-	air sacculitis, colibacillosis	GI-19	MW877627

IBV/Korea/18/2018	South Korea	2018-02-07	PS[a)]	-	air sacculitis, urate deposition	GI-19	MW877628
IBV/Korea/22/2018	South Korea	2018-02-23	Broiler	22 days	urate deposition	GI-19	MW877629
IBV/Korea/25/2018	South Korea	2018-03-08	Broiler	16 days	colibacillosis	GI-19	MW877630
IBV/Korea/61/2018	South Korea	2018-04-17	Broiler	18 days	urate deposition	GI-19	MW877631
IBV/Korea/76/2019	South Korea	2019-02-27	Broiler	24 days	nephritis, urate deposition	GI-19	MW877632
IBV/Korea/150/2019	South Korea	2019-05-23	Broiler	29 days	air sacculitis	GI-19	MW877633
IBV/Korea/144/2018	South Korea	2018-10-10	Broiler	28 days	colibacillosis, nephritis, urate deposition	GI-19	MW877634
IBV/Korea/151/2018	South Korea	2018-10-18	Broiler	-	nephritis, air sacculitis	GI-19	MW877635
IBV/Korea/151/2019	South Korea	2019-05-27	Layer	-	colibacillosis, urate deposition	GI-19	MW877636
IBV/Korea/193/2018	South Korea	2018-12-27	Broiler	21 days	colibacillosis, nephritis	GI-19	MW877637
IBV/Korea/60/2018	South Korea	2018-04-17	Broiler	21 days	urate deposition	GI-19	MW877638
IBV/Korea/23/2018	South Korea	2018-02-28	Broiler	24 days	colibacillosis, urate deposition	GI-19	MW877639
IBV/Korea/24/2018	South Korea	2018-02-28	Broiler	-	colibacillosis, nephritis	GI-19	MW877640
IBV/Korea/28/2018	South Korea	2018-03-13	Broiler	26 days	nephritis, urate deposition	GI-19	MW877641
IBV/Korea/59/2016	South Korea	2016-03-22	Broiler	20 days	nephritis, urate deposition	GI-19	MW877642
IBV/Korea/064/2018	South Korea	2018-04-24	Broiler	18 days	air sacculitis	GI-19	MW877643
IBV/Korea/17/2018	South Korea	2018-02-08	Broiler	35 days	nephritis, urate deposition	GI-19	MW877644
IBV/Korea/63/2016	South Korea	2016-03-30	Layer	40 days	respiratory signs	GI-19	MW877645
IBV/Korea/37/2017	South Korea	2017-03-22	Layer	27 weeks	respiratory signs, air sacculitis	GI-19	MW877646
IBV/Korea/76/2017	South Korea	2017-04-30	Broiler	22 days	nephritis, urate deposition	GI-19	MW877647
IBV/Korea/113/2017	South Korea	2017-09-16	Broiler	-	respiratory signs, urate deposition	GI-19	MW877648
IBV/Korea/33/2017	South Korea	2017-02-11	Broiler	24 days	nephritis, urate deposition	GI-19	MW877649
IBV/Korea/85/2016	South Korea	2016-04-21	Broiler	28 days	respiratory signs	GI-19	MW877650
IBV/Korea/87/2016	South Korea	2016-04-26	Broiler	27 days	no exact record	GI-19	MW877651

IBV/Korea/4/2016	South Korea	2016-01-13	Broiler	32 days	respiratory signs, nephritis	GI-19	MW877652
IBV/Korea/73/2016	South Korea	2016-04-08	Broiler	28 days	air sacculitis	GI-19	MW877653
IBV/Korea/166/2016	South Korea	2016-07-29	Broiler	22 days	nephritis	GI-19	MW877654
IBV/Korea/80/2016	South Korea	2016-04-19	Layer	10 weeks	no exact record	GI-19	MW877656
IBV/Korea/148/2019	South Korea	2019-05-16	PS	14 days	urate deposition	GI-19	MW877657
IBV/Korea/5/2020	South Korea	2020-01-17	Layer	49 days	nephritis, urate deposition	GI-19	MW877658
IBV/Korea/97/2016	South Korea	2016-05-03	Broiler	25 days	nephritis	GI-19	MW877659
IBV/Korea/55/2017	South Korea	2017-04-05	Broiler	30 days	urate deposition	GI-19	MW877660
IBV/Korea/173/2017	South Korea	2017-10-22	Broiler	13 days	nephritis, urate deposition	GI-19	MW877661
IBV/Korea/177/2018	South Korea	2018-11-23	Broiler	23 days	colibacillosis, nephritis, urate deposition	GI-19	MW877662
IBV/Korea/51/2018	South Korea	2018-04-06	PS	3 weeks	nephritis	GI-19	MW877663
IBV/Korea/62/2018	South Korea	2018-04-18	Broiler	28 days	nephritis	GI-19	MW877664
IBV/Korea/181/2020	South Korea	2020-10-06	PS	35 weeks	decreased egg production	GI-19	MW877665
IBV/Korea/264/2019	South Korea	2019-11-12	Broiler	22 days	urate deposition	GI-19	MW877666
IBV/Korea/269/2019	South Korea	2019-11-12	Broiler	22 days	colibacillosis	GI-19	MW877667

a) Parents stock.

Table S2. GI-19 reference strains used in this study.

Virus	Nation	Year	Genbank accession number
58HeN_93II	China	1993	KC577395
43SD-96III	China	1996	KC577385
36SD-97II	China	1997	KC577380
CK/CH/LLN/98I	China	1998	DQ167145
CK/CH/LHLJ/99I	China	1999	DQ167142
LS2	China	2002	AY278246
CK/CH/LXJ/02I	China	2002	DQ167152
CK/CH/LSD/03L	China	2003	DQ167148
K1019/03	SouthKorea	2003	FJ807927
K1255/03	SouthKorea	2003	FJ807928
NL/L_1449T/04	Europe	2004	EF079116
GB_552/04	Europe	2004	JF900374
GB_1011/04	Europe	2004	JF900375
L-1148	Europe	2004	DQ431199

K1583/04	SouthKorea	2004	FJ807931
K283/04	SouthKorea	2004	FJ807923
K463/04	SouthKorea	2004	FJ807924
gammaCoV/Ck/Poland/548/2004	Europe	2004	KT886436
CK/CH/LHLJ/04V	China	2004	DQ167139
CK/CH/LHLJ/04V	China	2004	FJ821752
CK/CH/LJL/04I	China	2004	DQ167144.
CK/CH/LHLJ/04XI	China	2004	DQ167140
FR/L_1450L/05	Europe	2005	EF079117
FR/L_1450T/05	Europe	2005	EF079118
GB_313/05	Europe	2005	JF900376
GB_1291/05	Europe	2005	JF900378
gammaCoV/Ck/Poland/29/2005	Europe	2005	KT886437
ITA/90254/2005	Europe	2005	FN182281
GB_1215/05	Europe	2005	JF900377
QIA-KR/D79/05	SouthKorea	2005	KU900740
CK/CH/LHLJ/05I	China	2005	EF213560
CK/CH/LHLJ/05VI	China	2005	EF213565
GX_NN4	China	2005	JX436329
CK/CH/JS/06I	China	2006	EU031525
GB_650/06	Europe	2006	JF900381
GB_210/06	Europe	2006	JF900379
gammaCoV/Ck/Poland/14/2006	Europe	2006	KT886438
GB_218/06	Europe	2006	JF900380
gammaCoV/Ck/Poland/1612/2006	Europe	2006	KT886440
HH06	China	2006	EF577030
CK/CH/LLN/06I	China	2006	EF213566
R2_2/99	China	2006	FJ829872
GB_429/07	Europe	2007	JF900382
GB_891/07	Europe	2007	JF900383
GB_892/07	Europe	2007	JF900384
UK/AV2150/07	Europe	2007	EU914939
IBVSX2	China	2007	FJ793938
IBVSX8	China	2007	FJ793940
IBVSX9	China	2007	GQ853589
DY07	China	2007	HM245923
HF2	China	2007	JX080381
GB_464/08	Europe	2008	JF900387
GB_1141/08	Europe	2008	JF900389
GB_1262/08	Europe	2008	JF900390
GB_1428/08	Europe	2008	JF900391
GB_733/08	Europe	2008	JF900388
IBV/La/SP/17/08	Europe	2008	GQ253482
IBV/Ck/SP/18/08	Europe	2008	GQ253483
IBV/Ck/SP/79/08	Europe	2008	GQ253484
A2_2	China	2008	HM031117
KD_08	China	2008	GQ844990

S_03	China	2008	HM031118
GB_171/09	Europe	2009	JF900392
GB_1237/09	Europe	2009	JF900394
GB_1574/09	Europe	2009	JF900395
GB_1646/09	Europe	2009	JF900396
gammaCoV/Ck/Poland/74/2009	Europe	2009	KT886454
QX_like	Europe	2009	JN022548
IBV/Ck/SP/170/09	Europe	2009	GQ253486
IBV/La/SP/116/09	Europe	2009	GQ253485
IBV/chicken/UK/3355/09	Europe	2009	HM132098
ck/CH/LHLJ/090712	China	2009	HM194666
ck/CH/LNX/090442	China	2009	HM194683
CK/CH/HB/HC09-2	China	2009	HQ018884
WZL	China	2009	HM034814
83GX_09V	China	2009	KC577409
80GX_09IV	China	2009	KC577407
GX_YL0902	China	2009	JX292014
GB_839/10	Europe	2010	JF900397
GB_1084/10	Europe	2010	JF900398
GB_1835/10	Europe	2010	JF900401
GB_1247/10	Europe	2010	JF900399
QX_like	Europe	2010	JN022545
QX_like	Europe	2010	JN022553
QX_like	Europe	2010	JN022550
gammaCoV/Ck/Poland/G024/2010	Europe	2010	KT886441
CK_CH_GX_GL1009	China	2010	KC692281
ck/CH/LSD/100505	China	2010	JF330889
1114	SouthKorea	2010	JQ920383
CK/CH/SD10/002	China	2010	JF738084
YX10	China	2010	JX840411
GX_GL	China	2010	JX292008
SCCQ_1	China	2010	JF951367
SCZJ_3	China	2010	JF951370
CK/CH/SCDY/10I	China	2010	HM106336
SCZJ_1	China	2010	JF951368
GX_YL1121	China	2010	JX292006
102GD_10II	China	2010	KC577422
GX_YL1002	China	2010	JX292010
gammaCoV/Ck/Poland/G033/2011	Europe	2011	KT886442
11039/11	SouthKorea	2011	JQ920401
11045/11	SouthKorea	2011	JQ920403
11038/11	SouthKorea	2011	JQ920400
11036/11	SouthKorea	2011	JQ920399
11035/11	SouthKorea	2011	JQ920398
CK/CH/HuB/HC11	China	2011	KJ524589
CK/CH/JS/YC11_2	China	2011	KJ524588
GX_NN_4	China	2011	JX291983

GX_NN_11	China	2011	JX291988
GX_GL11077	China	2011	JX291992
CK/CH/FJ/PT11	China	2011	KJ524586
GX_NN_9	China	2011	JX291987
ck/CH/IBYZ/2011	China	2011	KF663561
CK/CH/GX/NN11_1	China	2011	KJ524582
CK/CH/YN/SL12	China	2012	KJ524632
CK/CH/GD/LZ12	China	2012	KJ524638
SDIB702/2012	China	2012	KF007204
SDIB768/2012	China	2012	KF007205
SC1202	China	2012	KC478592
SDIB764/2012	China	2012	KF007207
SDIB764/2012	China	2012	KJ469742
SDIB778/2012	China	2012	KJ469744
SC1204	China	2012	KC478590
CK/CH/2013/QL1301	China	2013	KU361189
CK/CH/2013/TM33	China	2013	KU361190
CK/CH/2013/TM66	China	2013	KU361191
SDIB876/2013	China	2013	KJ469746
CK/CH/2013/XZ151	China	2013	KU361192
CK/CH/2013/XZ153	China	2013	KU361193
CK/CH/2014/QL1401	China	2014	KU361196
CK/CH/2014/QL1402	China	2014	KU361197
gammaCoV/ck/China/I0336/16	China	2016	MH427432
gammaCoV/ck/China/I0607/16	China	2016	MH427444
gammaCoV/ck/China/I1116/16	China	2016	MH427450
gammaCoV/ck/China/I0502/17	China	2017	MH427462
gammaCoV/ck/China/I0506/17	China	2017	MH427468
gammaCoV/ck/China/I0503/17	China	2017	MH427463
